# Supplementary figures and images for: Enhanced mosquito vectorial capacity underlies the Cape Verde Zika epidemic
Source: PLoS Biol. 2022 Oct 26;20(10):e3001864. doi: 10.1371/journal.pbio.3001864 (PMC9604947; doi:10.1371/journal.pbio.3001864)

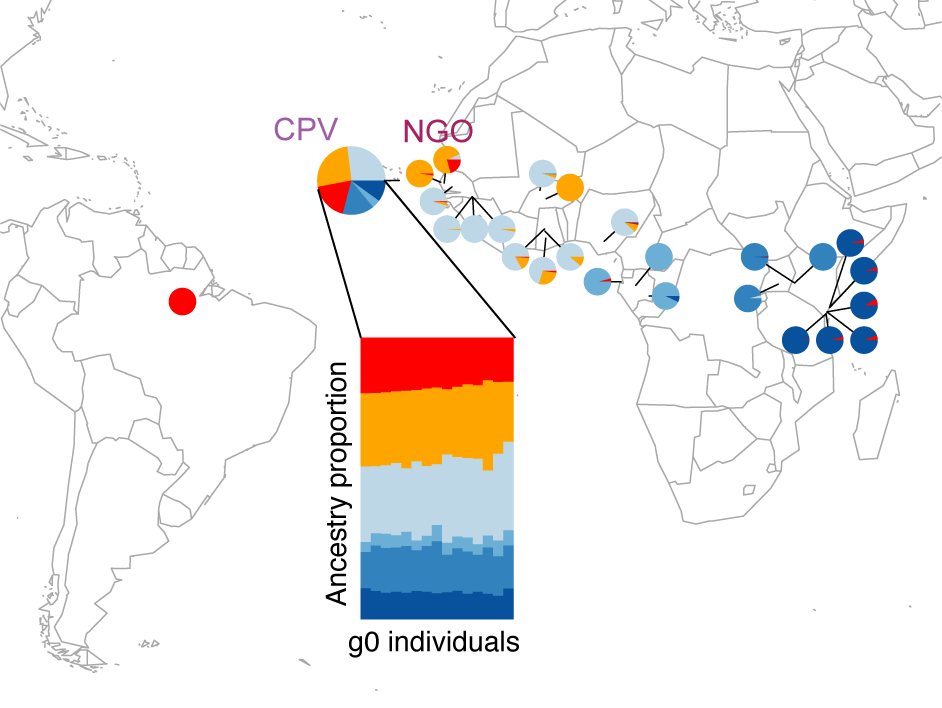

Supplement: S1 Fig — We previously found that when specifying 6 ancestry components (K = 6), ADMIXTURE identified 2 distinct human-specialist ancestry components—a putatively ancestral African human-specialist component (marked in orange) and a globally invasive component corresponding to human-specialist lineages that left Africa and spread across the global tropics (marked in red) [24]. Human-specialist ancestry in Cape Verde includes substantial contributions from each of these components—this may reflect gene flow from both the invasive range (likely Brazil) and the west coast of Africa (likely Senegal and/or Angola). However, more extensive sampling from both the Americas and native range would be necessary to conclusively identify precise source populations. The base layer of the map is from https://urldefense.com/v3/__https://cran.r-project.org/web/packages/maps/index.html__;!!JFdNOqOXpB6UZW0!sDKfbulNRVtMqxn5q2Db55SN93DxP6cPYdDUDB-Xy-GHDAIhO8GAhHf5FEJstypYbq4eF3PhXs9wPufm5y4eFCa5$. (TIF) [file pbio.3001864.s001.tif]
