# Supplementary material for: Enhanced mosquito vectorial capacity underlies the Cape Verde Zika epidemic
Source: PLoS Biol. 2022 Oct 26;20(10):e3001864. doi: 10.1371/journal.pbio.3001864 (PMC9604947; doi:10.1371/journal.pbio.3001864)
Supplement: S1 Text — (DOCX) [file pbio.3001864.s002.docx]

**Experimental procedures**

**Mosquitoes**

Wild *Ae. aegypti* eggs were collected in December 2020 using ovitraps deployed in the municipality of Praia, Santiago Island, which was the epicenter of the 2015-2016 Zika epidemic (1). About 500 generation 0 (g0) eggs were shipped to Institut Pasteur in Paris where they were hatched to establish a laboratory colony. First-generation (g1) eggs from this colony were sent to Princeton University, where approximately 500 individuals were amplified through an additional generation before being subjected to host preference assays. All experimental infections and behavioral assays took place within two generations of laboratory colonization. Mosquitoes were maintained under controlled insectary conditions (28°C, 12h:12h light:dark cycle and 70% relative humidity at Institut Pasteur; 26°C, 12h:12h light:dark cycle and 75% relative humidity at Princeton University). Larvae were reared in plastic trays containing 1.5 liters of tap water and supplemented with a standard diet of Tetramin (Tetra) fish food at a density of 200 larvae per tray. After emergence, adults were kept in 30 × 30 × 30 cm BugDorm-1 insect cages (BugDorm) with continuous access to 10% sucrose solution. For colony maintenance, mosquitoes were blood fed on commercial rabbit blood (BCL) at Institut Pasteur and on human arms at Princeton University. Mosquito colonies from Senegal, Gabon and Guadeloupe whose susceptibility to ZIKV was previously characterized (2) were included as references in the infection experiments. Mosquito colonies from Uganda, Florida and Senegal whose human preference was previously characterized (3) were included as references in the host preference assays.

**Genomic analysis**

To test for human-specialist ancestry in *Ae. aegypti* samples from Cape Verde, the genomes of 15 g0 individuals (10 females and 5 males) collected as eggs were re-sequenced. DNA was extracted by first disrupting whole mosquito tissues using a TissueLyser II set to 25 Hz for 2 min, and then proceeding with Qiagen DNeasy spin-column extractions for each individual. DNA was quantified using a Qubit 2.0 fluorometer, and unique-dual-index libraries were constructed for each individual using the tagmentation-based Illumina DNA Prep protocol. Individuals were sequenced to 20× average depth (2,532,280,212 total reads, range of 11×-29× across individuals) using a NovaSeq 6000 instrument with S4 chemistry.

Illumina adapter sequences were removed (clipping settings 2:3:10) and the read quality was controlled (leading :3, trailing :3, and minimum read length of 36) using Trimmomatic (4). Trimmed reads were mapped to the African consensus genome based on the AaegL5 assembly using *BWA-MEM*, as previously described (3,5). *PicardTools* was used to remove duplicates (<https://broadinstitute.github.io/picard/>). Individual genotypes were called with *bcftools* (6) at the unlinked set of 1 million single-nucleotide polymorphism (SNP) sites previously used to characterize population structure in (3). Analysis with *VCFTOOLS relatedness2* confirmed that none of the 15 samples were siblings (7). A total of 3,185 SNP sites that were no longer biallelic after incorporating the Cape Verde samples were set to missing, and ADMIXTURE was used to re-calculate ancestry proportions using as input the same population allele frequency estimates that were obtained in (3) at K=3 (8). This yielded estimates of ancestry proportions from three ancestry components previously identified in (3) – human-specialist, West African generalist, and East/Central African generalist.

**Host preference assays**

Preference for human hosts was measured with a two-port live-host olfactometer as previously described (3). Briefly, 24h before each trial, approximately 100 female mosquitoes were placed into a 32-oz plastic holding chamber, where they were starved of sucrose but provided with water on cotton balls. Before each trial, mosquitoes were allowed to acclimatize to the main olfactometer chamber for 5 min. Carbon-filtered, conditioned air fed from an external source was then provided at a rate of 0.6 m/s, passing through chambers containing either a guinea pig or a human elbow sealed at the forearm and biceps with a silicone gasket. The guinea pig was provided an absorbent pad to stand on, and an absorbent pad was also included on the human side to control for any effects of pad odor. After the start of each trial, mosquitoes were given 10 min to seek either human or guinea pig odor. After flying into a trap leading to either odor source, mosquitoes were not able to return to the main olfactometer chamber and were prevented from biting hosts by a mesh barrier. Mosquitoes that chose human, guinea pig, or did not respond were subsequently counted in a 4°C cold room.

Guinea pigs (two females in alternation) were used as the non-human hosts because of their good disposition and history of use in the host preference literature. Previous studies have shown that guinea pigs can be used to reliably differentiate human-specialist and generalist populations of *Ae. aegypti*, and that results are very similar to those obtained using different non-human odor sources (e.g., quail, African grass rat, laboratory rat, chicken, sheep’s wool, dog hair) (3,9–11). Similarly, although humans differ in relative attractiveness to *Ae. aegypti*, human-specialist populations reliably prefer a range of humans over guinea pigs and other non-human hosts (3,10,12). The present study involved the same 31-year-old European-American male human host as for the main behavioral trials described in (3).

Behavioral trials were carried out across 4 days, testing the same four *Ae. aegypti* colonies each day: the Cape Verde colony (CPV, g2); a generalist colony from Zika, Uganda (ZIK, g13); a human-specialist laboratory colony from Orlando, Florida (ORL); and a human-specialist colony from Ngoye, Senegal (NGO, g7). Testing order was randomized within each day and the side that human and non-human hosts were presented on was alternated across days, such that each of the two guinea pigs was used once on each side. Differences in host preference among laboratory colonies were tested with beta-binomial generalized linear mixed models (GLMMs) as implemented in the R package *glmmTMB*, including a random factor for trial day (13,14). The 95% confidence interval for the probability of seeking the human host was constructed using the R package *emmeans* (15).

**Experimental infections**

Experimental mosquito infections used two wild-type ZIKV strains with less than 5 passages in cell culture (2). The Cambodia 2010 ZIKV strain (FSS13025) was obtained from the World Reference Center for Emerging Viruses and Arboviruses at the University of Texas Medical Branch. The Senegal 2011 ZIKV strain (Kedougou2011) was obtained from the Institut Pasteur in Dakar. The Cambodia 2010 strain and the Senegal 2011 strain were chosen to represent the Asian and the African lineages of ZIKV, respectively (16). High-titered viral stocks were prepared and their infectious titers were measured by standard focus-forming assay as previously described (17). A commercial mouse anti-flavivirus group antigen monoclonal antibody (MAB10216; Merck Millipore) diluted 1:1,000 in phosphate-buffered saline (PBS; Gibco Thermo Fisher Scientific) supplemented with 1% bovine serum albumin (BSA; Interchim) was used as the primary antibody. The secondary antibody was an Alexa Fluor 488-conjugated goat anti-mouse antibody (A-11029; Life Technologies) diluted 1:500 in PBS supplemented with 1% BSA.

Mosquitoes were orally challenged with ZIKV as previously described (18). Briefly, 3- to 7-day-old females deprived of sucrose solution for 18h were offered an artificial infectious blood meal for 15 min using a Hemotek membrane-feeding apparatus (Hemotek Ltd.) with porcine intestine as the membrane. Blood meals consisted of a 2:1 mix of washed human erythrocytes and virus suspension, supplemented with ATP (Merck) at a final concentration of 10 mM. To establish the dose responses, the mosquitoes were exposed to different virus concentrations by diluting the virus stocks in cell culture medium prior to preparing the artificial infectious blood meal. Fully engorged females were sorted on wet ice and maintained under controlled conditions (28°±1°C, 12h:12h light:dark cycle and 70% relative humidity) in a climatic chamber with continuous access to 10% sucrose solution. After 7 days of incubation, the mosquito bodies were homogenized individually and ZIKV RNA was detected by RT-PCR as previously described (2).

The ZIKV susceptibility of four mosquito colonies was tested simultaneously: the Cape Verde colony (CPV, g2); a reference ZIKV-resistant colony from Lopé, Gabon (g21); a reference ZIKV-susceptible colony from Saint François, Guadeloupe (g17); and a colony from Ngoye, Senegal (NGO, g9) with intermediate ZIKV susceptibility. To test differences in ZIKV susceptibility between colonies, the proportion of infected mosquitoes was analyzed as a function of the infectious dose (log_10_-transformed blood meal titer) by logistic regression in JMP version 10.0.2 ([www.jmpdiscovery.com](http://www.jmpdiscovery.com)). The 50% oral infectious dose (OID_50_) values and their respective 95% confidence intervals were derived from the logistic fits.

**References**

1. Faye O, de Lourdes Monteiro M, Vrancken B, Prot M, Lequime S, Diarra M, et al. Genomic epidemiology of 2015–2016 Zika virus outbreak in Cape Verde. Emerg Infect Dis. 2020;26(6):1084.

2. Aubry F, Dabo S, Manet C, Filipović I, Rose NH, Miot EF, et al. Enhanced Zika virus susceptibility of globally invasive Aedes aegypti populations. Science. 2020;370(6519):991–6.

3. Rose NH, Sylla M, Badolo A, Lutomiah J, Ayala D, Aribodor OB, et al. Climate and Urbanization Drive Mosquito Preference for Humans. Curr Biol. 2020;30(18):3570-3579.e6.

4. Bolger AM, Lohse M, Usadel B. Trimmomatic: a flexible trimmer for Illumina sequence data. Bioinformatics. 2014;30(15):2114–20.

5. Li H. Aligning sequence reads, clone sequences and assembly contigs with BWA-MEM. arXiv:13033997. 2013.

6. Li H. A statistical framework for SNP calling, mutation discovery, association mapping and population genetical parameter estimation from sequencing data. Bioinformatics. 2011;27(21):2987–93.

7. Danecek P, Auton A, Abecasis G, Albers CA, Banks E, DePristo MA, et al. The variant call format and VCFtools. Bioinformatics. 2011;27(15):2156–8.

8. Alexander DH, Novembre J, Lange K. Fast model-based estimation of ancestry in unrelated individuals. Genome Res. 2009;19(9):1655–64.

9. Gouck HK. Host preferences of various strains of Aedes aegypti and A. simpsoni as determined by an olfactometer. Bull World Health Organ. 1972;47(5):680.

10. McBride CS, Baier F, Omondi AB, Spitzer SA, Lutomiah J, Sang R, et al. Evolution of mosquito preference for humans linked to an odorant receptor. Nature. 2014;515(7526):222–7.

11. Zhao Z, Zung JL, Hinze A, Kriete AL, Iqbal A, Younger MA, et al. Mosquito brains encode unique features of human odour to drive host seeking. Nature. 2022;605(7911):706-712.

12. DeGennaro M, McBride CS, Seeholzer L, Nakagawa T, Dennis EJ, Goldman C, et al. orco mutant mosquitoes lose strong preference for humans and are not repelled by volatile DEET. Nature. 2013;498(7455):487–91.

13. Brooks ME, Kristensen K, van Benthem KJ, Magnusson A, Berg CW, Nielsen A, et al. glmmTMB balances speed and flexibility among packages for zero-inflated generalized linear mixed modeling. R J. 2017;9(2):378–400.

14. R Core Team. R: A language and environment for statistical computing. 2013.

15. Lenth R, Singmann H, Love J. Emmeans: Estimated marginal means, aka least-squares means. R Package. 2018.

16. Aubry F, Jacobs S, Darmuzey M, Lequime S, Delang L, Fontaine A, et al. Recent African strains of Zika virus display higher transmissibility and fetal pathogenicity than Asian strains. Nat Commun. 2021;12(1):1–14.

17. Fontaine A, Jiolle D, Moltini-Conclois I, Lequime S, Lambrechts L. Excretion of dengue virus RNA by Aedes aegypti allows non-destructive monitoring of viral dissemination in individual mosquitoes. Sci Rep. 2016;6(1):1–10.

18. Baidaliuk A, Miot EF, Lequime S, Moltini-Conclois I, Delaigue F, Dabo S, et al. Cell-fusing agent virus reduces arbovirus dissemination in Aedes aegypti mosquitoes in vivo. J Virol. 2019;93(18):e00705-19.
