# Supplementary material for: Enhanced mosquito vectorial capacity underlies the Cape Verde Zika epidemic
Source: PLoS Biol. 2022 Oct 26;20(10):e3001864. doi: 10.1371/journal.pbio.3001864 (PMC9604947; doi:10.1371/journal.pbio.3001864)
Supplement: Attachment — Submitted filename: Rose review 2022 cape verde.docx [file pbio.3001864.s004.docx]

The manuscript, ‘Enhanced mosquito vectorial capacity underlying the Cape Verde Zika epidemic’ describes results from experimental evaluation of *Ae. aegypti* derived from Cape Verde designed to test the hypothesis that populations that facilitated ZIKV outbreaks possess *aegypti aegypti* genetic signature, associated with increased human biting preference and ZIKV competence. The studies are straight-forward, and results are not necessarily unexpected, yet they provide novel and important data that clearly support this hypothesis. This work has broad implications for understanding factors that facilitate transmission of Aedes-borne pathogens on the African continent and beyond. The manuscript is well-written, and the experimental design/interpretation are sound, yet there are some minor points that should be considered.

Broad considerations:

- Mosquito population used for experiments. Authors refer to the CPV colony but do not detail how this colony was derived. Are these mosquitoes directly from the field or, if not, how many generations have they been in the laboratory? If they are colonized do they retain the reported genetic signature?
- In the virus infection studies there is just one population of ‘pure’ formosus (Gabon). I wonder how representative this phenotype is among formosus? There is likely to be some variability among populations that could be important. The data is sufficient to support the main point of the manuscript, but some discussion is warranted.
- Has the virus associated with the Cape Verde outbreak in 2015-16 been isolated and/or sequenced? There is limited discussion regarding the potential role of viral genotype in outbreaks.
- Viral dissemination. There are likely to be differences in viral dissemination rates among populations that may be distinct from infection rates. If the authors removed legs from experimentally infected mosquitoes, they should consider testing and reporting these results.

Minor suggested edits:

- Line 200- delete ‘besides’
- Line 201- delete ‘or failure’
