# Supplementary material for: Enhanced mosquito vectorial capacity underlies the Cape Verde Zika epidemic
Source: PLoS Biol. 2022 Oct 26;20(10):e3001864. doi: 10.1371/journal.pbio.3001864 (PMC9604947; doi:10.1371/journal.pbio.3001864)
Supplement: Attachment — Submitted filename: Rose_et_al_response_to_reviewers.docx [file pbio.3001864.s005.docx]

**Point-by-point response to Reviewers**

*Lines numbers refer to the track-changes version of the revised manuscript.*

**Reviewer #1:**

The manuscript, ‘Enhanced mosquito vectorial capacity underlying the Cape Verde Zika epidemic’ describes results from experimental evaluation of *Ae. aegypti* derived from Cape Verde designed to test the hypothesis that populations that facilitated ZIKV outbreaks possess *aegypti aegypti* genetic signature, associated with increased human biting preference and ZIKV competence. The studies are straight-forward, and results are not necessarily unexpected, yet they provide novel and important data that clearly support this hypothesis. This work has broad implications for understanding factors that facilitate transmission of Aedes-borne pathogens on the African continent and beyond. The manuscript is well-written, and the experimental design/interpretation are sound, yet there are some minor points that should be considered.

Broad considerations:

- Mosquito population used for experiments. Authors refer to the CPV colony but do not detail how this colony was derived. Are these mosquitoes directly from the field or, if not, how many generations have they been in the laboratory? If they are colonized do they retain the reported genetic signature?

*Response* : We apologize if this was unclear. The CPV colony was initiated from wild *Ae. aegypti* eggs collected using ovitraps deployed in the municipality of Praia, Santiago Island, which was the epicenter of the 2015-2016 Zika epidemic. About 500 wild eggs were shipped to Institut Pasteur in Paris where they were hatched to establish a laboratory colony. First-generation eggs from this colony were sent to Princeton University, where approximately 500 individuals were amplified through an additional generation before being subjected to host preference assays. All mosquito experiments (both infection and behavioral assays) took place within two generations of laboratory colonization. We have added a sentence in the main text on lines 160-162 to clarify this point. All the methodological details are provided in the SI.

- In the virus infection studies there is just one population of ‘pure’ formosus (Gabon). I wonder how representative this phenotype is among formosus? There is likely to be some variability among populations that could be important. The data is sufficient to support the main point of the manuscript, but some discussion is warranted.

*Response* : We consider the population from Gabon to be representative of *Aaf* based on our previous study (Aubry et al., 2020). All the ‘pure’ *Aaf* populations (<10% *Aaa* ancestry) had similar levels of ZIKV susceptibility (OID_50_ values within one log_10_ unit). We have added text on lines 181-184 to address this point.

- Has the virus associated with the Cape Verde outbreak in 2015-16 been isolated and/or sequenced? There is limited discussion regarding the potential role of viral genotype in outbreaks.

*Response* : Viruses isolated during the Cape Verde epidemic were sequenced and the phylogenetic analysis indicated that an Asian strain was introduced from the Americas (Faye et al., 2020). ZIKV consists of two lineages, the Asian and the African lineages. All Zika outbreaks to date have been associated with strains from the Asian lineage, although ZIKV strains from the African lineage have a higher epidemic potential in laboratory studies (Aubry et al., 2021). We have added text on line 74 and lines 95-96 to expand our discussion of the role of viral genotype.

- Viral dissemination. There are likely to be differences in viral dissemination rates among populations that may be distinct from infection rates. If the authors removed legs from experimentally infected mosquitoes, they should consider testing and reporting these results.

*Response* : We did not collect and test the legs of mosquitoes to assess viral dissemination, however we know from our earlier study (Aubry et al., 2020) that higher infection rates are associated with enhanced potential to transmit ZIKV from a viremic host in generalist *Ae. aegypti*. We have added text on lines 206-208 to clarify this point.

Minor suggested edits:

- Line 200- delete ‘besides’
- Line 201- delete ‘or failure’

*Response* : We have made these corrections.

**Reviewer #2:**

This manuscript describes the higher vector competence of Aedes aegypti mosquitoes from Cape Verde to Zika viruses and their higher preference to blood feed on humans with respect to other African mosquitoes. This report appears to be a simple complement of a recent work authored by the same first author (Rose et al., 2020).
While I agree that preference for humans is a factor that can contribute to higher vector competence of Cape Verde mosquitoes, I do not agree on the (exclusive) emphasis put on it on this manuscript. Please see below main comments, which will hopefully help authors to provide a less skewed and more comprehensive hypothesis on the occurrence of the 2015-2016 Zika outbreak in Cape Verde.

*Response*: We strongly agree that human preference is just one of several factors that can contribute to vectorial capacity and is not at all the exclusive driver of arboviral spread. We thank the Reviewer for the helpful comments below – we have incorporated them and hope that the revised manuscript provides a more balanced picture.

Lines 57-60. This sentence is exaggerated and negates all the work done by scientists as Ross and McDonald. Factors that influence the emerge and spread of vector-borne disease, including arboviruses, have well been understood and codified into the vectorial capacity equation. These factors include vector density and extrinsic incubation period besides vector competence, which appears to be the focus of this manuscript. I would agree that factors that influence vector competence are still poorly characterised and that preference to feed on humans influences the blood-feeding rate, which is another parameter already codified into the standard equation of vectorial capacity produced by Ross and MacDonald.

*Response*: We did not intend to neglect the work of Ross and MacDonald and have revised this paragraph to make our point more accurately. Although the concept of vectorial capacity has certainly contributed to understand how mosquitoes spread arboviruses in general, we believe the widespread geographical disparities in arbovirus emergence and spread are still incompletely understood. We have added the following sentence on lines 51-54, explicitly pointing to a review of Ross and MacDonald’s work: “The overall ability of mosquitoes to spread arboviruses is well described by the concept of vectorial capacity, which mathematically codifies the key entomological parameters driving transmission (Smith et al., 2012).”

Lines 85-99. Are Aedes species other than Ae. aegypti found in Cape Verde, mimicking a similar situation as in Yap?

*Response*: We have clarified on lines 119-120 that *Ae. aegypti* is the only known ZIKV vector species found in Cape Verde – however *Ae. caspius*, which is not thought to spread ZIKV, has been documented there.

Line 101-103 and 187-189. The Sahel region is quite extensive and ranges from Senegal, Mali, Burkina Faso, Nigeria, Cameroon, Central African Republic, Chad and Ethiopia. African populations of Ae. aegypti that were analysed and found to be human specialists by Rose et al. 2020 are limited to few populations in Senegal, to my knowledge. These populations are also cited in this ms (line 142). Ae. aegypti populations from Kenya and Angola have a completely different genetic ancestry than those from Senegal as described by several papers from the group of Jeffrey Powell, thus cannot be equated to human-specialist populations from Senegal.

Response: We thank the Reviewer for bringing up this important point – we have clarified throughout the text that we meant the West African Sahel, drawing on work done in Senegal and Burkina Faso. We agree that the Kenyan and Angolan populations are quite different with distinct ancestries and histories – we have clarified on lines 108-110 that what they have in common is an “*Aedes aegypti aegypti*”-like genomic signature.

Line 158-162: In this experiment, mosquitoes from Cape Verde showed stronger preference to seek humans than the Aaf strain from Uganda, as shown in Fig. 1B. However, in the supplementary methods Rose et al. described the use of 31 European-American male human individuals as host to perform the behavioural trials, while no African human individuals were included, not even from Cape Verde. Although the evaluation on humans preference from Africa and out of Africa mosquitoes is not strictly one of the main goals of this study, they did not mention whether there is any biological reason suggesting that the strong human preference observed in Fig. 1B will not change regardless of the human host's geographical origin and/or whether such preference will vary with African people.

*Response*: To address this point, we have indicated on lines 168-172 that previous research (including supplementary data provided in Rose et al., 2020) has found that the documented patterns do not depend on the ethnicity or sex of the human host used.

Lines 185-187. I agree with the authors that preference for humans and high intrinsic susceptibility to Zika virus of mosquitoes from Cape Verde with respect to Aaf populations are important factors that could have contributed to the Cape Verde ZIKV epidemics of 2015-2016, but also the density of both hosts and vectors are important. Any information on abundance and distribution of vectors in Cape Verde and human density? Please discuss.

*Response*: This is indeed a relevant point and we have expanded our discussion on lines 215-218: “Other factors such as human population density and mosquito abundance could also play an important role in driving differences in the spread of ZIKV; however, Praia is a relatively small city of about 150,000 inhabitants where the recorded mosquito abundances are not unusually high (Campos et al., 2020).”

Line 202-208. Among the populations analysed by Rose et al. 2020, there is a Brazilian population and this hypothesis could be easily tested. In the ancestry plot shown in Fig. 1, how much is the ancestry linking Cape Verde with Brazil vs other Ae. aegypti populations tested in Rose et al. 2020?

*Response*: We thank the Reviewer for pointing this out – we have performed additional ADMIXTURE analyses and provided a new supplementary figure (S1 Figure) documenting likely gene flow from both invasive populations outside of Africa (likely Brazil) and human-specialist populations from West Africa. However, more extensive sampling from both the Americas and the native range would be necessary to conclusively identify precise source populations.

**Reviewer #3:**

This manuscript aims to answer the question, why have large-scale Zika virus outbreaks not occurred in Africa. The authors provide provocative evidence to suggest that the underlying genetics of Aedes aegypti in Africa may be playing an important role. Aedes aegypti originated in Africa where it still persists today as a forest dwelling form (Aedes aegypti formosus) and an urban form (Aedes aegypti aegypti). As Aedes aegypti aegypti spread through the equatorial tropics it has become a predominately human-biting mosquito. However, in most of Africa, Aedes aegypti populations contain significant formosus genetic signatures. Aedes aegypti formosus has more catholic feeding preferences and has been shown to have reduced vector competence to Zika virus compared to Aedes aegypti aegypti populations from the Americas (for example). Interestingly, Aedes aegypti from Cape Verde, Africa —which experienced a large-scale ZIKV outbreak in 2015-2016—have a substantial Aedes aegypti aegypti genetic ancestry. The authors show that mosquitoes derived from Cape Verde populations have a stronger preference for human host odors similar to other human-specialist populations of Aedes aegypti. In addition, they show that mosquitoes derived from Cape Verde populations have significantly higher Zika virus infection rates than mosquitoes with different genetic backgrounds from Africa. All of which could portend future risk of Zika virus outbreaks for the African continent due to urbanization and expansion of mosquitoes with a similar genetic background as those found in Cape Verde. This manuscript is written well and the results mostly support the conclusions. In addition, this serves as an interesting foundation for extensive follow-up work.

My primary concern is with the title of the manuscript and the title to figure 1. It is inaccurate to state that mosquitoes from Cape Verde have "enhanced vectorial capacity" for Zika virus. Vectorial capacity is the basic reproductive rate of a vector-borne pathogen. Vectorial capacity and vector competence cannot be used interchangeably. Vectorial capacity takes into account all of the environmental, behavioral, cellular, and biochemical factors that influence the association between a vector, the pathogen transmitted by the vector, and the vertebrate host to which the pathogen is transmitted. Here, the authors assessed host preference and mosquito vector competence—two components of overall vectorial capacity but these are not sufficient to quantitatively determine whether one population of mosquitoes has enhanced vectorial capacity compared to another population.

*Response*: We agree with the Reviewer that vector competence and host preference are only two components of overall vectorial capacity, however any increase in vector competence and/or human preference (assuming other parameters are constant) inevitably augments vectorial capacity. We have expanded our discussion on lines 215-218 to acknowledge the potential influence of other parameters influencing vectorial capacity: “Other factors such as human population density and mosquito abundance could also play an important role in driving differences in the spread of ZIKV; however, Praia is a relatively small city of about 150,000 inhabitants where the recorded mosquito abundances are not unusually high (Campos et al., 2020).” Following the Editor’s suggestion, we have only slightly modified the title to ‘Enhanced mosquito vectorial capacity underlies the Cape Verde Zika epidemic’.

In addition, some text should be devoted to justify using infection rates as a proxy for vector competence and limitations relevant to the random and inefficient nature of mosquito transmission. Indeed, it has been previously shown that Aedes aegypti with poor competence but high population density have been capable of sustaining outbreaks of arboviral diseases like yellow fever virus. Also, infection data are only from a single timepoint, why was day 7 chosen?

*Response*: We focused on infection rates measured on day 7 for consistency with our earlier study (Aubry et al., 2020), which showed that higher infection rates are associated with enhanced potential to transmit ZIKV from a viremic host in generalist *Ae. aegypti*. We have added text on lines 206-208 to clarify this point. We agree that low vector competence can be compensated by high mosquito densities, however, as noted above, the mosquito abundances that have been recorded in Praia are not unusually high (Campos et al., 2020).”
